# Celiac disease and hepatitis B virus: Celiac disease and HBV

**Published:** 2011-01-01

**Authors:** Margherita Bonamico, Maurizio Mennini, Laura Petrarca, Raffaella Nenna

**Affiliations:** 1 Department of Pediatrics Sapienza University, Rome, Italy

**Keywords:** Celiac, Hepatitis B virus

Dear Editor,

We read with interest the article by Leonardi and La Rosa (2010) that deals with the hypothesis that nonintestinal inflammatory diseases, such as hepatitis B virus (HBV), may trigger immunologic gluten intolerance in susceptible people [1]. A recent study indicated that a high frequency of rotavirus infections may increase the risk of celiac disease (CD) autoimmunity in genetically predisposed individuals [2]. Although we recognize the relevance of this controversial field of study, we cannot accept that a sample size of 60 patients is sufficient to explain this epidemiological link. We would like to offer some interesting considerations regarding the transmission of HBV in Italy. Survey data from Italy, where a universal vaccination program was started in 1991 for infants as well as adolescents, showed a clear overall decline in the incidence of acute hepatitis B cases from 11/100,000 in 1987 to 1.6/100,000 in 2006. This decline was even more striking in people between the ages of 15 and 24 years, for whom the morbidity rate per 100,000 fell from 17 in 1990 to less than 0.5 in 2006 [3]. Despite these substantial decreases in the incidence of HBV infection in Italy, during a similar time period (1991-2010) the number of children diagnosed with CD remained steady; specifically, in our own salivary screening of 5,000 primary-school children, the prevalence of CD reached 1.3% [4]. This suggests that the decrease of HBV infection, which was a result of Italy's careful immunization program, did not influence the presumed incidence of CD (1%). Finally, as we know, the gold standard for the diagnosis of CD is a small-intestine biopsy because of the limits of serological screening. A workshop [5] that aimed to evaluate the concordance and improve the effectiveness of tissue transglutaminase autoantibodies (AbtTG) assays among various research and clinical laboratories demonstrated that the sensitivity levels of ELISA and radiobinding assays reached 91% and 93%, respectively, suggesting a particularly high sensitivity in detecting low-titer sera. Therefore, using AbtTG ELISA as a screening tool, it is possible that low-titer-positive adults could have CD but be undiagnosed. The data provided by Leonardi and La Rosa [1] are not sufficient to either hypothesize or refute a link between HBV and CD.
